# Heterogeneity of breast cancer stem cells as evidenced with Notch-dependent and Notch-independent populations

**DOI:** 10.1002/cam4.18

**Published:** 2012-07-18

**Authors:** Nelson K Y Wong, Megan Fuller, Sandy Sung, Fred Wong, Aly Karsan

**Affiliations:** 1 Genome Sciences Centre, BC Cancer AgencyVancouver, BC, Canada; 2 Department of Pathology and Laboratory Medicine, University of British ColumbiaVancouver, BC, Canada; 3 Cancer Genetics Laboratory, BC Cancer AgencyVancouver, BC, Canada

**Keywords:** Breast cancer, cancer stem cells, dominant-negative MAML, MCF-7, Notch

## Abstract

Studies have suggested the potential importance of Notch signaling to the cancer stem cell population in some tumors, but it is not known whether all cells in the cancer stem cell fraction require Notch activity. To address this issue, we blocked Notch activity in MCF-7 cells by expressing a dominant-negative MAML-GFP (dnMAML) construct, which inhibits signaling through all Notch receptors, and quantified the effect on tumor-initiating activity. Inhibition of Notch signaling reduced primary tumor sphere formation and side population. Functional quantification of tumor-initiating cell numbers in vivo showed a significant decrease, but not a complete abrogation, of these cells in dnMAML-expressing cells. Interestingly, when assessed in secondary assays in vitro or in vivo, there was no difference in tumor-initiating activity between the dnMAML-expressing cells and control cells. The fact that a subpopulation of dnMAML-expressing cells was capable of forming primary and secondary tumors indicates that there are Notch-independent tumor-initiating cells in the breast cancer cell line MCF-7. Our findings thus provide direct evidence for a heterogeneous cancer stem cell pool, which will require combination therapies against multiple oncogenic pathways to eliminate the tumor-initiating cell population.

## Introduction

In recent years, much effort has been invested into studying a subpopulation of tumor cells, termed cancer stem cells (CSC) [1]. These cells are hypothesized to initiate and sustain tumors through self-renewal as well as differentiation into transit-amplifying cells [1]. The transit-amplifying cells are thought to possess greater proliferative capacity, but lack the self-renewal capability, and thus cannot maintain tumors over time nor reinitiate tumors following therapy [2]. Initial studies demonstrated that transplantation of CD34^+^CD38^−^ human leukemic cells recapitulated the original leukemia in immunocompromised mice, but that other cell fractions did not [3]. Subsequent efforts identified CSC in solid tumors of various organs, including the breast and the brain [4, 5]. Although it has been argued that xenotransplantation of human cells into immunocompromised mice underestimates the CSC frequency [6, 7], mouse mammary tumor models do support the CSC paradigm [8–10].

*NOTCH* was originally identified in Drosophila where haploinsufficiency results in “notched” wing development [11]. The essential role of Notch signaling in cell fate decisions was subsequently identified in many tissues [12]. For mammary gland development, Notch signaling has been shown to promote human mammary stem cell expansion [13]. Transcriptome analysis of human tissue shows that dynamic changes of expression levels of Notch members and ligands are associated with various stages of mammary epithelial development [14]. In particular, *NOTCH3* was shown to be essential for bipotent progenitors to differentiate into luminal epithelial cells [14]. Intriguingly, Notch signaling seems to limit expansion of mouse mammary stem cells [15], which is in contrast to the observation in human mammary stem cells. However, in both systems, Notch signaling promotes differentiation toward luminal epithelial cells. Thus, Notch signaling is essential to the biology of mammary stem cells in mouse and human.

Aberrant activation of Notch signaling has long been implicated in breast cancer development. It was originally observed that one of the preferential integration sites of murine mammary tumor virus (MMTV) was Notch4 [16], leading to hyperactive Notch signaling [17]. Moreover, expression of constitutively active Notch4 in mice, controlled by the whey acidic protein promoter, produced mammary tumors [18]. Furthermore, human breast epithelial cells can be transformed through ectopic expression of active Notch4 [17]. In addition, accumulation of the cytoplasmic domain of Notch1 was apparent in many breast cancer cell lines and tumor tissues, indicative of active Notch1 signaling [19]. Grudzien et al. showed that blockade of Notch signaling, through pharmacological reagents or Notch1 knockdown, inhibited sphere formation from breast cancer cell lines. The result suggests that Notch signaling may be essential to self-renewal of CSC [20]. It has also been shown that Notch4 signaling is more active in CSC-enriched population [21]. Moreover, inhibition of Notch function decreases in vivo tumorigenicity [21]. However, how Notch signaling affects CSC frequency has not yet been determined.

To address the requirement of Notch on CSC function, we chose the human breast cancer cell line, MCF-7, as it has been shown to be a CSC-driven cell line by different groups [22, 23]. It has been demonstrated that MCF-7 cells cultured in suspension as spheres [22] or selected by the side population [23] enriches the CSC population with significantly higher potential for tumor initiation. To block Notch signaling, the dominant-negative MAML-GFP (dnMAML) construct [24] was employed. The dnMAML construct was developed with the C-terminus truncation of mastermind-like 1, which is an essential coactivator of Notch signaling [25]. The interaction of dnMAML with CSL and the cytoplasmic domain of Notch is thought to competitively inhibit binding of endogenous mastermind-like proteins and prevent downstream transcriptional activation [24]. Thus, dnMAML is a pan-Notch signaling inhibitor, and it has been used to demonstrate Notch-dependence in various systems, including growth dependence of T-cell acute lymphoblastic leukemia [25], the fate decision of lymphocyte development in vivo [24], and vascular smooth muscle cell differentiation in vivo [26].

## Materials and Methods

### Animals and cells

Female NOD-SCID mice were purchased in-house from the Animal Resources Centre at the BC Cancer Research Centre. The mice were between the ages of 6–10 weeks at the time of estrogen pellet and cancer cell implantation. Pellets containing 1.5 μg of the estradiol-17β with either 60-day or 90-day release (Innovative Research of America, Sarasota, FL) were implanted 1 day before the subcutaneous injection of cancer cells. All animal procedures were approved by the Animal Care Committee at the University of British Columbia.

MCF-7 cells were a gift from Dr. J. Emerman (University of British Columbia, Vancouver, BC) and were authenticated by Genetica (Cincinnati, OH). SVEC4-10 is a mouse endothelial cell line derived from SV40 transformation [27], and these cells were retrovirally transduced to overexpress human Jagged-1 or YFP alone for the coculture experiments [28]. All cell lines were kept in DMEM containing 10% heat-inactivated fetal bovine serum, supplemented with 2 mM l-glutamate, streptomycin, and penicillin.

### Plasmids and viral infection

MSCV-GFP and MSCV-DNMAML1-GFP retroviral vectors have been previously described [24], and MCF7 cells were transduced according to previously described protocols [29].

### Coculture experiment

The experiment was carried out as previously described [28] with the following modifications. The coculture (MCF-7 and SVEC4-10) was seeded into 12-well plates at 1:1 ratio with 10^5^ cells each. After 48 h, the cells were lysed for protein or RNA extraction. Induction of Notch targets in the coculture experiment was compared between treatment with *γ*-secreatase inhibitor DAPT (Calbiochem, San Diego, CA) and carrier control DMSO (Sigma-Aldrich, St. Louis, MO).

### Western blotting

Cells were lysed in RIPA buffer and 50 μg of cell lysate was resolved by SDS-PAGE. The resolved protein was transferred to nitrocellulose membrane and probed with an anti-GFP antibody (Roche Diagnostics Corporation, Indianapolis, IN) or an antitubulin antibody (Sigma-Aldrich) followed by antimouse IgG-horseradish peroxidase (Sigma-Aldrich). The bands were visualized with chemiluminescence (Perkin Elmer, Woodbridge, ON) and exposure to X-ray film (Eastman Kodak Co., Rochester, NY).

### RT-qPCR

Reverse transcription was performed with 2.5 *μ*g of total RNA/sample with random primers and Superscript II (Invitrogen, Carlsbad, CA) according to the manufacturer's protocol. Subsequently, quantitative PCR was carried out using human-specific primers spanning exon–intron junctions. The sequences of the primers are reported in Table S2. Threshold cycles (Ct) of target transcripts were normalized against Ct of human GAPDH.

### Antibody labeling for flow cytometry analysis

MCF7-GFP and MCF7-dnMAML cells were trypsinized, enumerated, and resuspended in phosphate buffered saline containing 2% fetal bovine serum (flow medium). Two hundred thousand cells were incubated on ice for 30 min with flow medium alone or 1 μg (in 100μL) of anti-CD49f antibody (BD Pharmingen, San Diego, CA). After washing the cells once with the flow medium, the cells were incubated in the dark with 1:100 dilution of antirat IgG-Alexa 594 on ice for 30 min. Before flow cytometry analysis, the cells were washed twice and resuspended in the flow medium.

### Tumor sphere culture

Tumor sphere culture was initiated and kept in MammoCult (StemCell Technologies, Vancouver, BC) complete medium (containing growth supplement, heparin, and hydrocortisone) [30]. The initiation of the culture was done according to the manufacturer's protocol with slight modification. Generally, 2000–10,000 cells were seeded into each well of a 6-well low-adherence plate. Tumor spheres were counted 7 days after culture initiation and the number of tumor spheres was normalized as number of spheres per 100 cells seeded, designated as % sphere-forming unit.

### Side population/Hoechst exclusion assay

The experiment was carried out as previously described [22] with the following modifications: the cells were washed once with ice-cold HBSS before incubation with 1.5 μg/mL Hoechst 33342 (Sigma-Aldrich) with or without verapamil (Sigma-Aldrich); before flow cytometry analysis, labeled cells were resuspended in ice-cold HBSS/2% FBS.

### Flow cytometry analysis

Flow cytometry data were analyzed with WinMDI 2.9 (The Scripps Research Institute, San Diego, CA). Cells were first gated with forward scatter and time of flight. For MCF7-GFP and MCF-dnMAML cells, GFP^+^ cells were selected for analysis (using parental MCF-7 cells as nonfluorescent control). For CD49f staining, secondary antibody alone (anti-IgG-Alexa594) was used as nonfluorescent control. For Hoechst exclusion assay, cells treated with verapamil were used for gating the side population.

### Tumor implantation and calculation of tumor-doubling time

Cells were injected subcutaneously into the dorsal flank of anesthetized mice. For secondary implants, primary tumors were minced and digested with collagenase II (Sigma-Aldrich) and dispase (Invitrogen). Following DNAse (Sigma-Aldrich) treatment, cells were resuspended in ice-cold DMEM/20% fetal bovine serum and filtered through 100-*μ*m cell strainer. The cells were then centrifuged at 300 g and resuspended in DMEM/10% fetal bovine serum and sorted for GFP before subcutaneous implantation on the same day. Palpable tumors were measured and calculated as follows: Volume = Length × Width × Height × 0.523 and tumor-doubling time (in days) was calculated as described [31], using the formula log_10_(2)/slope of [log tumor volume vs. time (days)].

### Statistical analysis

Analyses were performed using Prism (Graphpad, La Jolla, CA) or Excel (Microsoft, Redmond, WA). As dnMAML was expected to have an inhibitory effect in all assays performed, one-tail tests were utilized. For calculation of CSC frequencies, web program of extreme limiting dilution was utilized (http://bioinf.wehi.edu.au/software/elda/) [32].

## Results

### Expression of dominant-negative MAML-GFP inhibits Notch signaling in MCF-7 cells, but does not alter growth of MCF-7 in vitro

**Figure 1:**
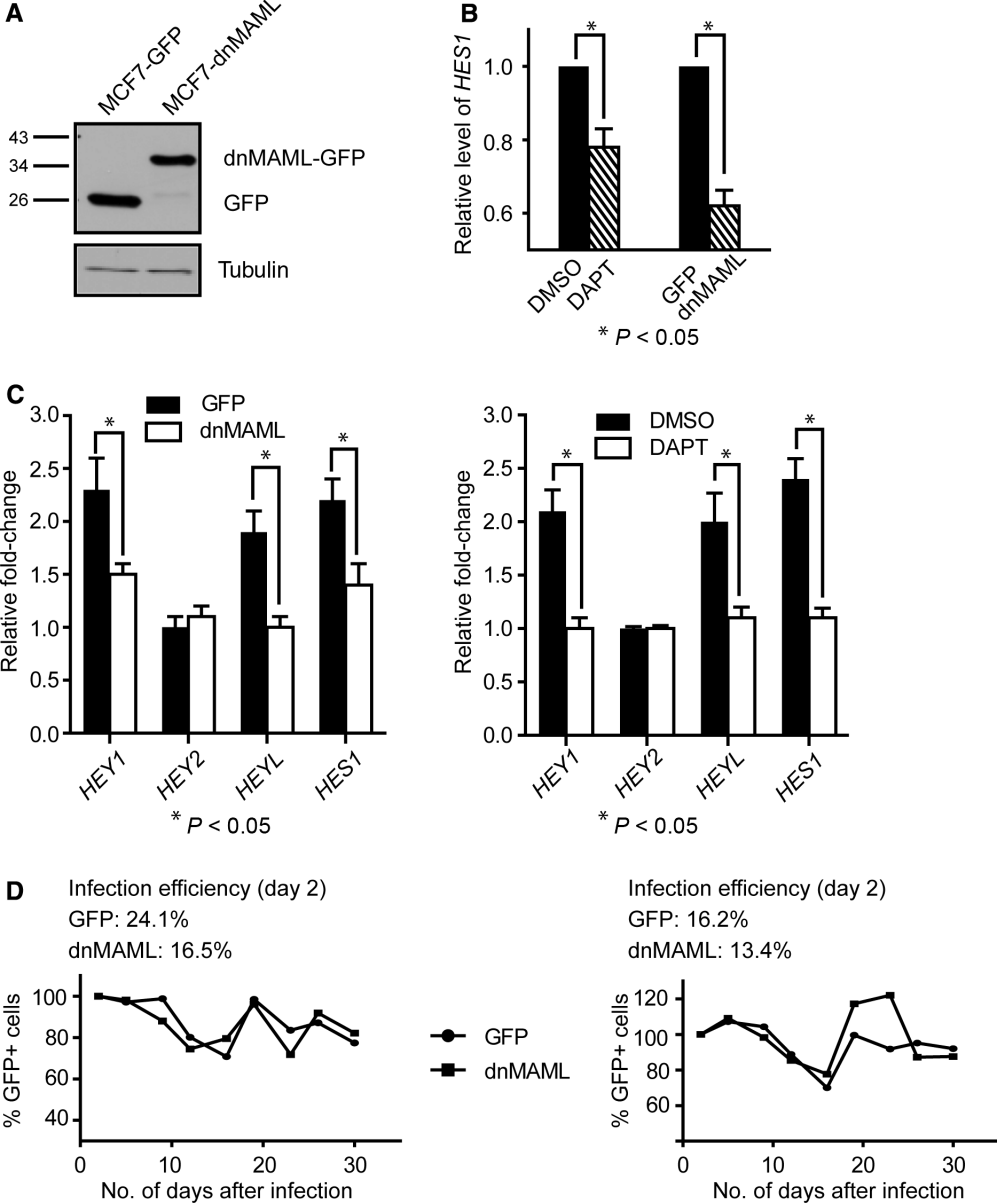
Expression of dnMAML in MCF-7 cells. (A) Western blot of cell lysate from MCF7-GFP and MCF7-dnMAML cells. The blot was probed with anti-GFP, stripped, and reprobed with antitubulin. (B) Comparison of *HES1* levels between DMSO- and DAPT-treated MCF-7 as well as comparison between MCF7-GFP and MCF7-dnMAML cells, as quantified with RT-qPCR. (C) Relative levels of *HEY* family members and *HES1* quantified with RT-qPCR in MCF-7 cells after 48 h of coculture. The expression levels were normalized to MCF-7 cells cocultured with SVEC-MIY cells (without Jagged-1 overexpression). Error bars in (B and C) are standard errors of the mean. (D) Percentage of GFP^+^ cells traced over a period of 30 days using flow cytometry. Results from two independent cell transductions are shown.

To block Notch signaling in MCF-7 cells, dominant-negative MAML-GFP (dnMAML) was overexpressed using a retroviral vector, whereas GFP alone was expressed as a control (Fig. 1A). Expression of the Notch target *HES1* was inhibited in the presence of dnMAML or the *γ*-secretase inhibitor DAPT (Fig. 1B).

To confirm that dnMAML inhibits ligand-induced Notch signaling in MCF-7 cells, we used a coculture system previously described [28]. MCF-7 cells expressing either GFP (vector control, MCF7-GFP) or dnMAML (MCF7-dnMAML) were cocultured with mouse SVEC4-10 cells expressing Jagged-1 or vector control. Using human-specific primers, Jagged-1-induced targets were assayed by RT-qPCR comparing MCF7-GFP/SVEC-YFP and MCF7-GFP/SVEC-Jag cocultures. *HEY1*, *HEYL*, and *HES1* were induced in coculture with Jagged-1 overexpressing cells, whereas *HEY2* was not induced. Expression of dnMAML or DAPT treatment inhibited the induction of *HEY1*, *HEYL*, and *HES1* in cocultured MCF-7 cells (Fig. 1C). Therefore, our results show that dnMAML blocks endogenous and Jagged-1-induced Notch signaling in MCF-7 cells.

To determine if expression of dnMAML conferred growth disadvantage on MCF-7 cells in standard tissue culture on plastic, cells were infected with the retroviral vectors containing either GFP or dnMAML and allowed to rest for 2 days. Then the proportion of GFP^+^ cells was followed for up to 30 days. Results from two independently made batches of cells showed that dnMAML did not affect the proliferation of MCF-7 cells plated on plastic (Fig. 1D).

### Blockade of Notch signaling decreases the proportion of CD49f^+^ cells, inhibits primary tumor sphere formation, and decreases side population cells

**Figure 2:**
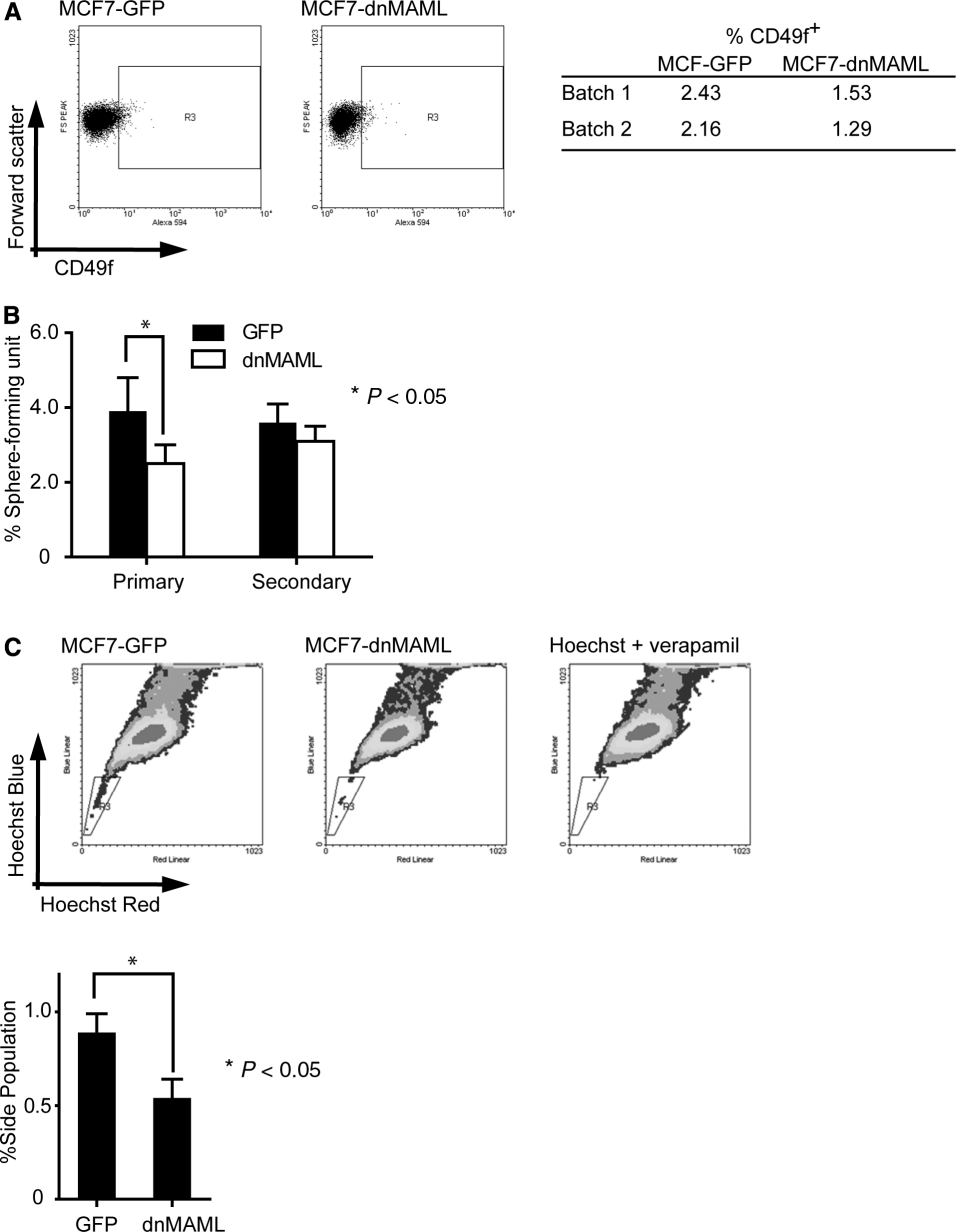
In vitro assessment of CSC population. (A) Percentage of CD49f^+^ cells in MCF-GFP and MCF-dnMAML cells as evaluated by flow cytometry. Results of two independently transduced batches of cells are shown. (B) Number of tumor spheres observed per 100 cells seeded, percentage of sphere-forming units, in primary (*P* = 0.029) and secondary tumor sphere culture (*P* = 0.15). At least three independent experiments using independent cell transductions were performed in each case. (C) Percentage of side population cells determined by Hoechst exclusion using flow cytometry (four independent experiments using three independent cell transductions, *P* = 0.027). Gating of side population was determined with verapamil inhibition. Error bars in (B and C) are standard errors of the mean.

To determine whether Notch blockade decreases the CSC population in MCF7-dnMAML cells, we first performed in vitro assays to examine changes in CSC populations. CD44^hi^/CD24^lo^ has been used to enrich CSC population in breast cancer cells [4]. Therefore, we stained MCF-7 cells with these two markers; however, we found that the proportion of CD44^hi^/CD24^lo^ cells was low (about 0.4%, Fig. S1). We also sought to use other breast cancer stem cell markers for evaluating CSC difference in MCF7-GFP and MCF7-dnMAML cells. Cariati et. al. have demonstrated that proportion of CD49f^+^ cells increases in the CSC-enriched fraction of MCF-7 cells and CD49f was required for tumor initiation [22]. We thus examined CD49f as a CSC marker in these cells. Results from flow cytometry analysis revealed that there was a decrease in the proportion of CD49f^+^ cells in MCF7-dnMAML cells when compared with MCF7-GFP. The decrease of CD49f^+^ cells in MCF7-dnMAML was consistently observed in two independently transduced batches of cells (Fig. 2A).

Dontu et. al. have described a serum-free and anchorage-independent assay for culturing mammary stem or progenitor cells [33]. Since then, many have utilized this system to study breast CSC and found that this culture system enriches the CSC population [9, 22, 30, 34]. Therefore, to quantify CSC with a functional assay, tumor sphere formation of MCF7-GFP and MCF7-dnMAML were evaluated. Both MCF7-GFP and MCF7-dnMAML cells were able to form primary sphere cultures in the serum-free and anchorage-independent conditions. However, the sphere-forming units of MCF7-dnMAML cells in primary culture were decreased by 40% when compared to MCF7-GFP cultures (Fig. 2B). Yet, no difference of sphere-forming units in secondary culture was observed. To determine if Notch signaling is active in the sphere culture, the expression levels of *HEY1* in monolayer and sphere cultures were compared. A consistent and significant increase in *HEY1* expression was observed in the sphere culture (Fig. S2A), and the induction was repressed by dnMAML expression or DAPT treatment (Fig. S2B).

As the side population of MCF-7 cells has been shown to enrich CSC [23], we performed Hoechst exclusion assays to evaluate the side populations of MCF7-GFP and MCF7-dnMAML cells. Our results show that MCF7-dnMAML cells possess a smaller side population (25% decrease) than that of the MCF7-GFP cells (Fig. 2C). Together, the smaller proportion of CD49f^+^ cells, lower capability in forming primary spheres, and the smaller side population of MCF7-dnMAML cells suggest that the CSC population is smaller in these cells.

### MCF7-dnMAML cells contain a lower frequency of cancer stem cells

**Figure 3:**
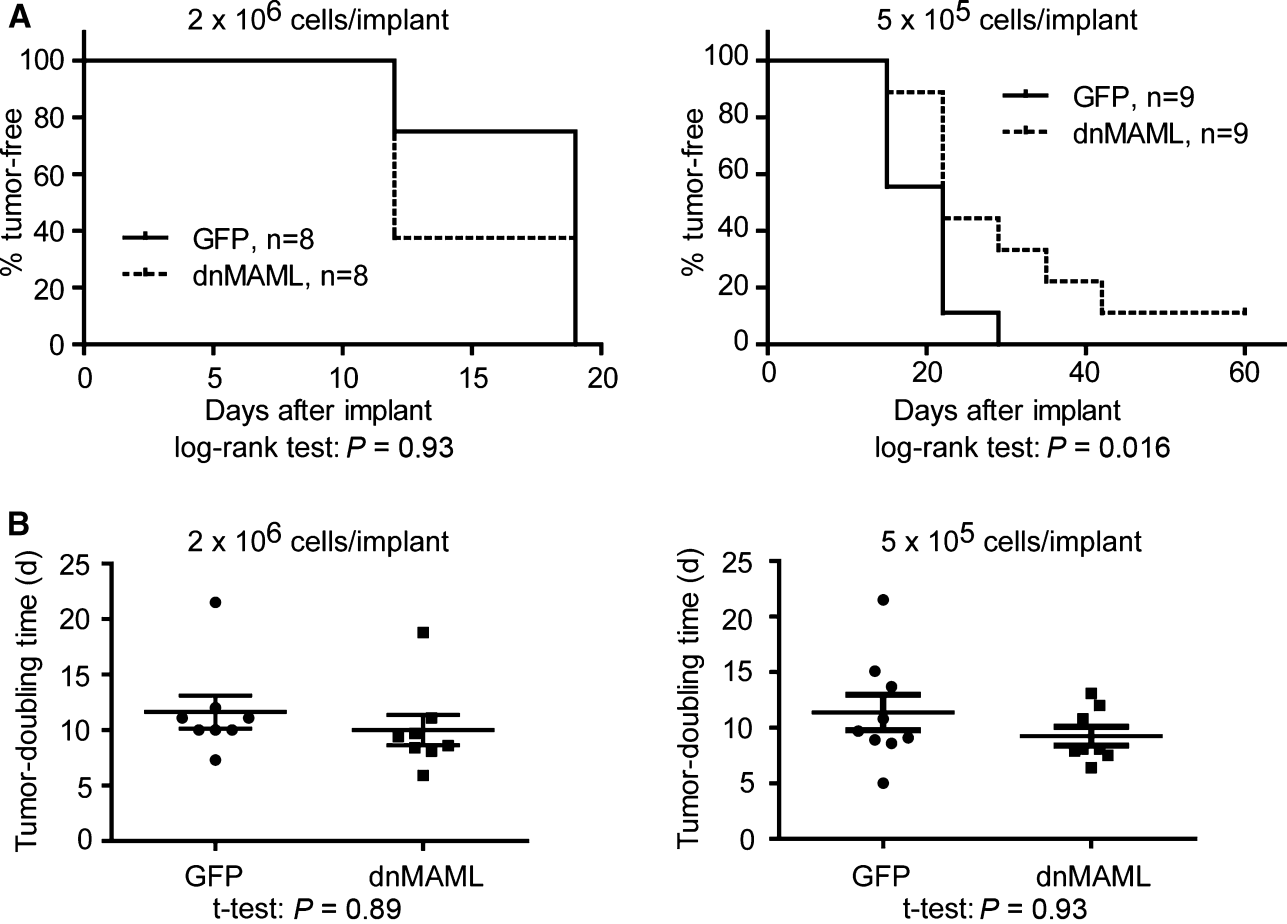
Detection of palpable tumors and growth characteristics of tumors over time. (A) When more cells (2 × 10^6^ cells/injection) were implanted, there was no significant difference between MCF7-GFP and MCF7-dnMAML cells as to when tumors were palpable. However, when fewer cells (5 × 10^5^ cells/injection) were implanted, tumors arising from MCF7-dnMAML cells showed a significant delay. (B) Tumor-doubling time with more cells (2 × 10^6^ cells/injection) or fewer cells (5 × 10^5^ cells/injection) implanted.

One major hallmark of CSC is the ability to initiate tumors in vivo. To determine the frequencies of CSC in MCF7-GFP and MCF7-dnMAML cells in vivo, we performed limiting-dilution xenograft experiments, and the mice were followed over a period of 90 days. The frequency of CSC was significantly less in MCF7-dnMAML populations, compared with MCF7-GFP cells, as calculated by extreme limiting dilution assay (Table 1) [32]. The estimated frequency of CSC in MCF7-GFP was 1 in 9748 cells, whereas that in MCF7-dnMAML was 1 in 24,489 cells (*P* = 0.013), showing a 60% decrease in CSC following Notch blockade. Moreover, MCF7-dnMAML cells showed a significant delay in the formation of palpable tumors when fewer cells were implanted (Fig. 3A). However, the delay in the appearance of palpable tumors was not associated with slower tumor growth, as suggested by the comparable tumor-doubling times (Fig. 3B). These results are compatible with an inhibition of a subset of CSC rather than the slowing of proliferation of all cells.

**Table 1:** Effect of Notch blockade on frequency of MCF-7 CSC assayed by limiting dilution

Types of cell implanted	No. of cells injected	No. of tumors observed/implant	CSC frequency (95% CI)
MCF7-GFP	2 × 10^6^	8/8	1/9748*
	2 × 10^4^	14/16	(1/15,527–1/6120)
	10^4^	8/11	
	4 × 10^3^	0/4	
MCF7-dnMAML	2 × 10^6^	8/8	1/24,489*
	2 × 10^4^	8/15	
	10^4^	4/9	(1/43,652–1/13,739)
	4 × 10^3^	0/4	

CSC, cancer stem cells.

* χ^2^ = 6.18, *P* = 0.013.

### Existence of Notch-independent CSC

Although expression of dnMAML decreased the CSC frequency, it was clear that MCF7-dnMAML cells were capable of forming tumors. To determine if the cells derived from MCF7-dnMAML tumors were capable of forming secondary tumors, GFP^+^ cells (MCF7-GFP or MCF7-dnMAML) were harvested from primary tumors and sorted for implantation. Twelve mice were implanted with MCF7-GFP cells implanted on the left flank and MCF7-dnMAML cells implanted on the right flank. For the MCF7-GFP cells, 7 of 11 implant sites formed tumors (1 excluded due to experimental error), whereas 5 of 12 MCF7-dnMAML implant sites formed secondary tumors, which is not significantly different (Fisher's exact test, *P* = 0.26).

To ensure that dnMAML expression was not lost in the secondary tumors, the proportion of GFP^+^ cells was examined in freshly isolated secondary tumors (Table S2). Cells derived from secondary tumors were also examined for GFP or dnMAML expression (Fig. S3A). Digested tumor cells were kept in culture for more than 2 weeks in MCF-7 growth medium. During that time, all the stromal cells died in the culture while the tumor-derived cancer cells reached purity of ∼99% GFP^+^ as examined by flow cytometry (Fig. S3A). Western blots of cell lysates showed that GFP or dnMAML was expressed in the tumor-derived cells (Fig. S3B). Therefore, our results demonstrate that Notch-inhibited MCF-7 cells derived from primary tumors are capable of reinitiating tumors at the same frequency as control tumors in serial assays, indicating that this subset of cells is capable of tumor initiation in a Notch-independent fashion.

## Discussion

Recent reports have shown that Notch signaling is essential for maintaining the CSC population in breast cancer cell lines. Harrison et al. showed that knocking down Notch4 decreased tumorigenicity of MCF-7 cells; however, the authors also suggested that their strategy did not distinguish between canonical and noncanonical Notch signaling [35]. Hoey and coworkers demonstrated lower CSC frequency with blocking DLL4 antibody [36]. Although the blocking antibody inhibits Notch signaling in OMP-C8 tumors, the possibility of the contribution of blocking DLL4 reverse signaling in CSC frequency cannot be denied [37].

We made use of a different strategy to block Notch signaling, that is, expression of dnMAML. As dnMAML has been shown to block signaling from all Notch receptors through its interaction with the intracellular domain of Notch and CSL [24], our observations emphasize the importance of Notch canonical signaling to a subpopulation of CSC. By blocking Notch signaling, a subpopulation of CSC was eliminated in the MCF-7 cells. This is suggested by the decrease in CD49f^+^ population, lower capability to form primary tumor spheres, and smaller side population, as these in vitro properties have all been demonstrated to be associated with CSC phenotype [9, 22, 23, 30, 34]. To date, definite surface marker or combination of surface markers for indentifying CSC is still lacking. The reported surface makers, including CD49f, CD44^hi^/CD24^lo^, and ESA, can only be used as a surrogate readout of CSC. The gold standard for evaluating CSC frequency is still the limiting dilution assay. The result of our in vivo limiting dilution assay shows that the CSC frequency in MCF7-dnMAML cells is lower, but not complete abrogation, when compared with that of the MCF7-GFP cells. This clearly demonstrates that Notch blockade is only essential to a subpopulation of CSC.

Although dnMAML-expressing cells showed a delay in forming palpable tumors, these tumors exhibited the same tumor-doubling time as those arising from GFP-expressing cells. This observation suggests that removal of Notch-dependent CSC affects the initial phase of tumor initiation, and once the tumors are initiated, the progression of the GFP and the dnMAML tumors are the same. Interestingly, when cells that had escaped Notch inhibition in the primary assay were reexamined in serial CSC assays, no difference was seen in the secondary tumor-initiating assay, suggesting again the emergence or coexistence of a Notch-independent CSC population. Our results suggest that there is heterogeneity in the tumor-initiating population with Notch signaling essential to only a subset of CSC. More experiments are still required to determine the signaling pathway(s) that regulates the Notch-independent pool of CSC. Nonetheless, our results suggest that it will be important in cancer treatment to use combination therapies even if the rationale is to treat the CSC population.

## Supporting Information

**Figure S1.** Flow cytometry analysis of breast cancer cell lines with putative CSC makers CD44 and CD24. MCF-7 or MDA-MB-231 cells were labeled with CD44-FITC (1:100, BD Pharmingen) and CD24-PE (1:100, BD Pharmingen) as described in Materials and Methods.

**Figure S2.** Relative expression levels of *HEY1*. RT-qPCR was performed as outlined in the Materials and Methods. The expression level of *HEY1*, a well-characterized gene regulated by Notch, was consistently and significantly upregulated in the sphere culture (A). The induction of *HEY1* expression is repressed by the expression of dnMAML or DAPT treatment in the sphere culture (B). The results were from at least three independent experiments and the error bars depict the standard error of the mean.

**Figure S3**. Flow cytometry analysis of freshly isolated tumor cells and cultured tumor cells. After the tumors were harvested, minced, and digested, cells from the tumors were analyzed by flow cytometry, using MCF-7 parental cells as a reference. All the tumors examined showed high proportion of GFP^+^ tumor cells (see Table S2). Cells derived from the tumors were kept in culture for 2 weeks, without sorting, before being analyzed again with flow cytometry. More than 99% of the cultured tumor-derived cells were GFP^+^. Three tumors from each of the GFP and dnMAML tumors were examined. Flow cytometry data were processed with WinMDI 2.9. (B) Western blot of lysate from cells arose in culture from GFP and dnMAML tumors. The blot was probed with anti-GFP.

**Table S1.** Primer sets used for reverse transcription-quantitative PCR.

**Table S2.** Percentage of GFP^+^ tumor cells at harvest.
