# Supplementary figures and images for: Heterogeneity of breast cancer stem cells as evidenced with Notch-dependent and Notch-independent populations
Source: Cancer Med. 2012 Jul 18;1(2):105–13. doi: 10.1002/cam4.18 (PMC3544441; doi:10.1002/cam4.18)

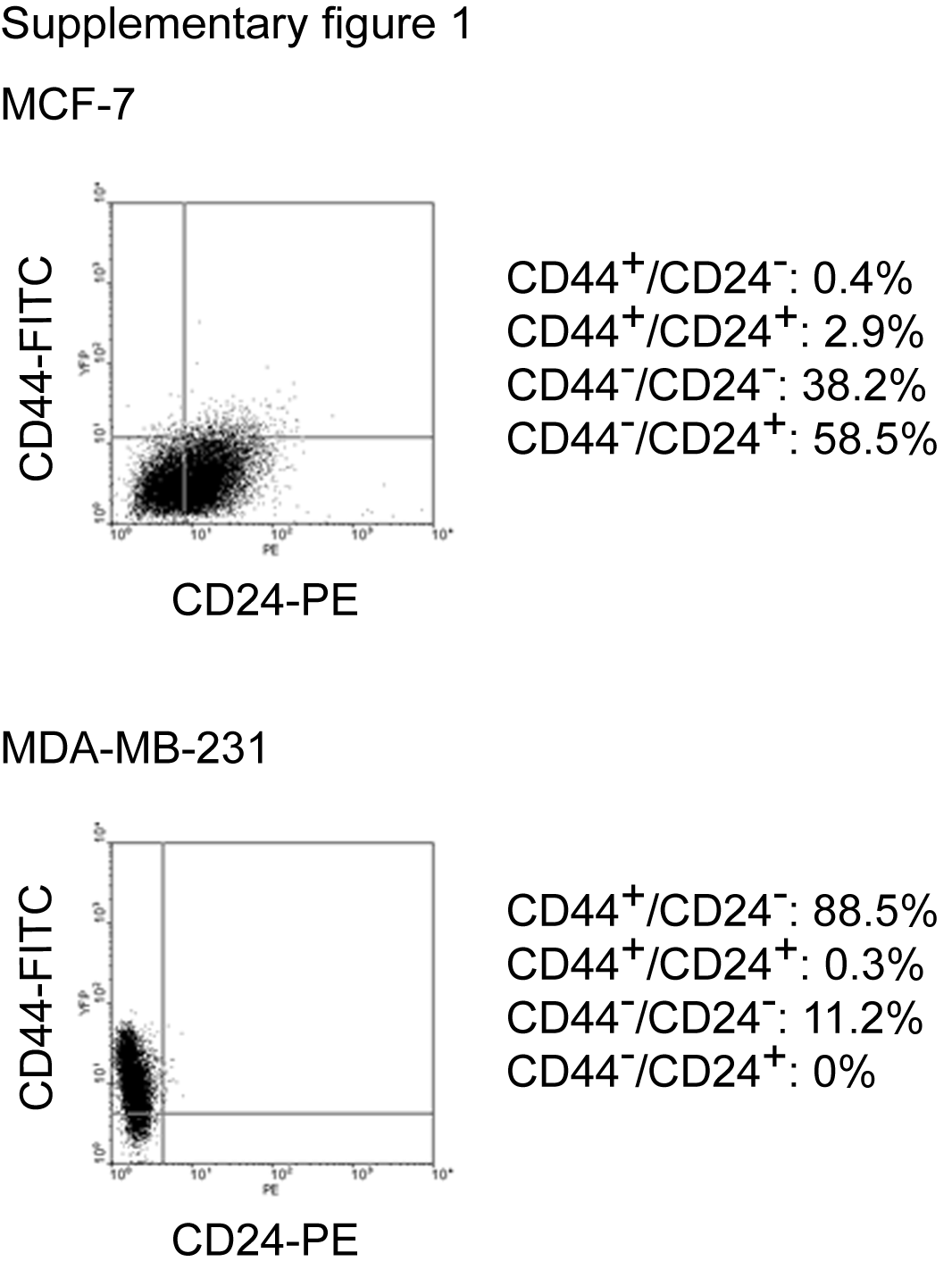

Supplement: Supplementary file 1 [file cam40001-0105-SD1.tif]

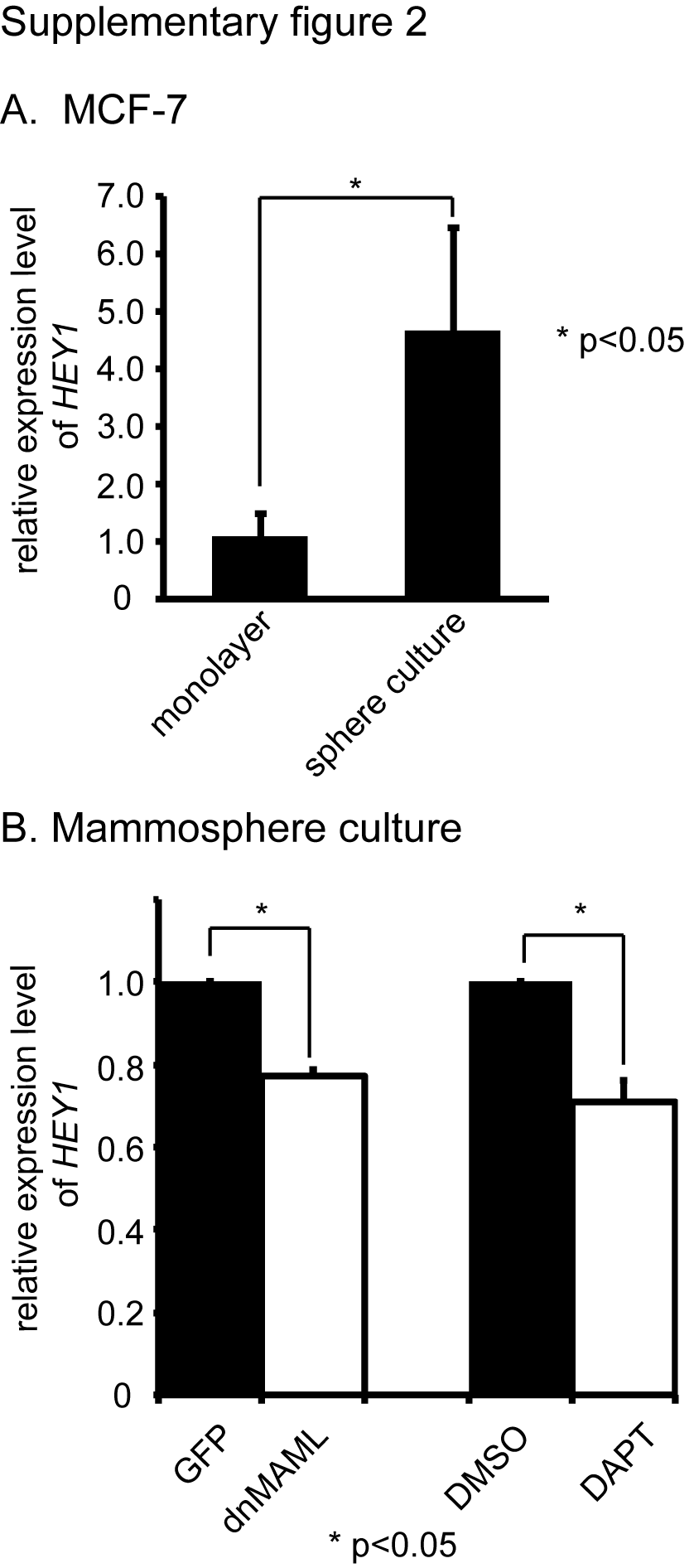

Supplement: Supplementary file 2 [file cam40001-0105-SD2.tif]

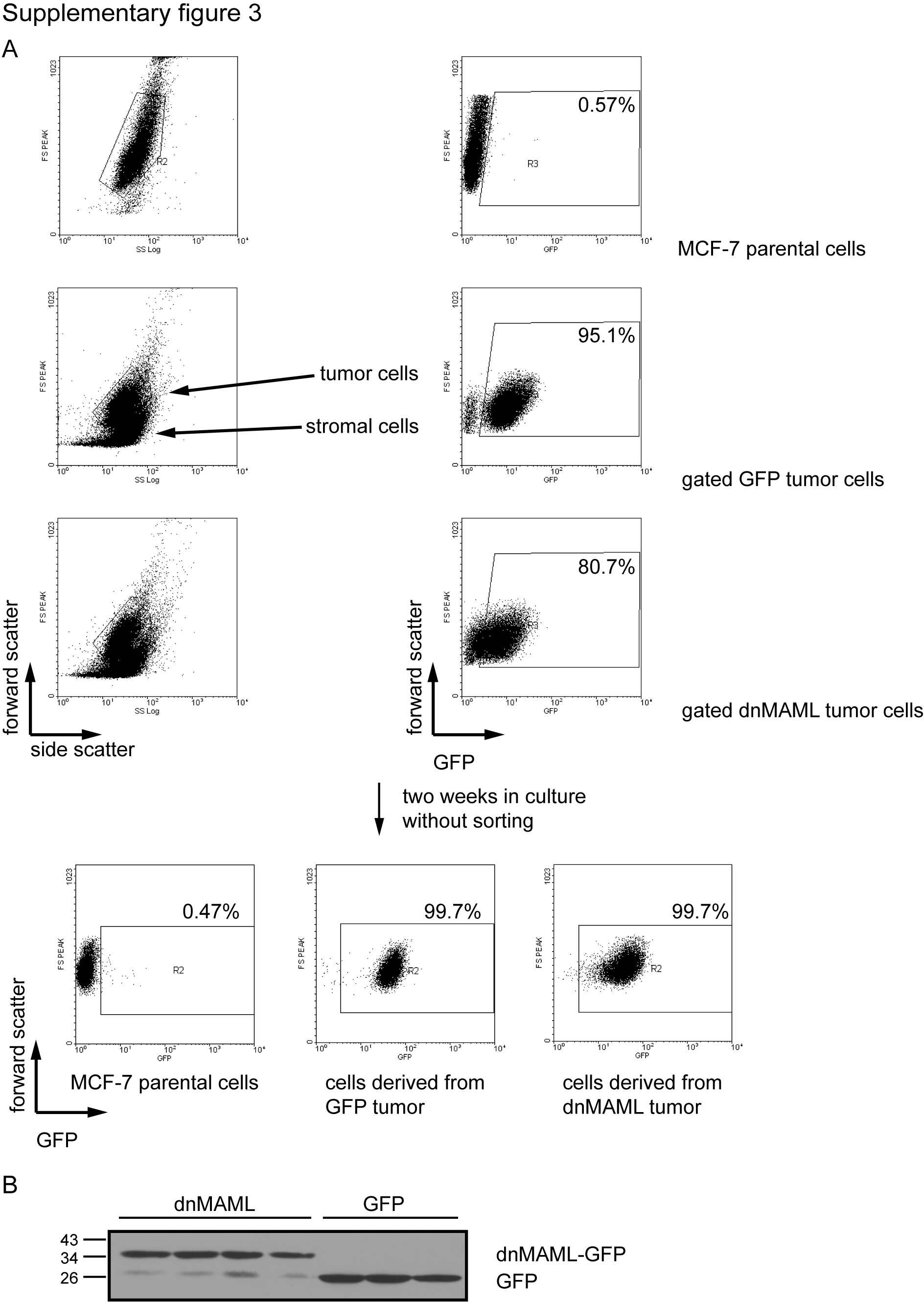

Supplement: Supplementary file 3 [file cam40001-0105-SD3.tif]
