# Supplementary material for: Heterogeneity of breast cancer stem cells as evidenced with Notch-dependent and Notch-independent populations
Source: Cancer Med. 2012 Jul 18;1(2):105–13. doi: 10.1002/cam4.18 (PMC3544441; doi:10.1002/cam4.18)
Supplement: Supplementary file 4 [file cam40001-0105-SD4.doc]

**Supplementary table 1**

Primer sets used for reverse transcription-quantitative PCR

| Gene | Forward primer (5’-3’) | Reverse Primer (5’-3’) |
| --- | --- | --- |
| GAPDH | GGACCTGACCTGCCGTCTAGAA | GGTGTCGCTGTTGAAGTCAGAG |
| Hey1 | AGAGTGCGGACGAGAATGGAAACT | CGTCGGCGCTTCTCAATTATTCCT |
| Hey2 | TTGAAGATGCTTCAGGCAACAGGG | TCAGGTACCGCGCAACTTCTGTTA |
| HeyL | ATGCAAGCCAGGAAGAAACGCAGA | AGCTTGGAAGAGCCCTGTTTCTCA |
| HES1 | CGACACCGGATAAACCAAAGACAG | CGCGAGCTATCTTTCTTCAGAGCA |
