# Supplementary material for: Heterogeneity of breast cancer stem cells as evidenced with Notch-dependent and Notch-independent populations
Source: Cancer Med. 2012 Jul 18;1(2):105–13. doi: 10.1002/cam4.18 (PMC3544441; doi:10.1002/cam4.18)
Supplement: Supplementary file 5 [file cam40001-0105-SD5.doc]

**Supplementary table 2**

Percentage of GFP^+^ tumor cells at harvest

| Tumor ID | % GFP^+^ tumor cells at harvest |
| --- | --- |
| GFP tumor 1 | 95.4% |
| GFP tumor 2 | 91.9% |
| GFP tumor 3 | 94.1% |
| dnMAML tumor 1 | 80.7% |
| dnMAML tumor 2 | 91.7% |
| dnMAML tumor 3 | 82.1% |
